# The Applicability of the Standard DIN EN ISO 3690 for the Analysis of Diffusible Hydrogen Content in Underwater Wet Welding

**DOI:** 10.3390/ma13173750

**Published:** 2020-08-25

**Authors:** Jan Klett, Thomas Wolf, Hans Jürgen Maier, Thomas Hassel

**Affiliations:** Institut für Werkstoffkunde (Materials Science), Leibniz Universität Hannover, An der Universität 2, 30823 Garbsen, Germany; wolf@iw.uni-hannover.de (T.W.); maier@iw.uni-hannover.de (H.J.M.); hassel@iw.uni-hannover.de (T.H.)

**Keywords:** diffusible hydrogen, SMAW, underwater wet welding, DIN EN ISO 3690, AWS A4.3, JIS Z 3113

## Abstract

The European standard ISO 3690 regulates the measurement of diffusible hydrogen in arc-welded metal. It was designed for different welding methods performed in dry atmosphere (20% humidity). Some details of the standard are not applicable for wet underwater welding. The objective of this study was to extend the applicability of DIN EN ISO 3690:2018-12 to underwater wet-shielded metal arc welding (SMAW). Four different aspects regulated within the standard were accounted for: (1) sample dimensions and number of samples taken simultaneously; (2) time limitations defined by the standard regarding the welding and the cleaning process; (3) time, temperature, and method defined for analysis of the diffusible hydrogen content; (4) normalization of the hydrogen concentration measured. Underwater wet welding was performed using an automated, arc voltage-controlled welding machine. The results are discussed in light of standard DIN EN ISO 3690, and recommendations are provided for the analysis of diffusible hydrogen content upon underwater wet welding.

## 1. Introduction

Due to the increasing number of offshore constructions in the wind and oil industry, the need to find appropriate ways to repair these buildings is the subject of many research projects. One of the key repair procedures for steel constructions ashore is the arc welding process, which can be transferred to underwater wet environments with some adaptations. However, as the process is transferred to the wet environment, the risk of cracks induced by diffusible hydrogen increases [1].

Hydrogen-induced cracking (HIC) occurs when the following three factors appear simultaneously [2]:

- Tensile stresses are present which approach the yield strength;
- The material features a microstructure that is susceptible to HIC;
- A critical amount of soluble hydrogen is present, which can diffuse within the lattice.

The steels used in modern offshore constructions often exceed an equivalent carbon content ($CEV\;\left(\%\right)=\frac{Mn\;\left(\%\right)}{6}+\frac{Cu\;\left(\%\right)\;+\;Ni\;\left(\%\right)}{15}+\frac{Cr\;\left(\%\right)\;+\;Mo\;\left(\%\right)\;+\;V\;\left(\%\right)}{5}$) of 0.4%; thus, there is a high risk that martensitic microstructures will develop in the heat-affected zone (HAZ) during underwater welding [3]. Welding can also result in residual tensile stress via constrained shrinkage. In wet conditions, the gas bubbles surrounding the arc can contain up to 92% hydrogen [4], which is partly dissociated by the arc. The absorption of hydrogen atoms by the melted or heated iron surface can, thus, rapidly result in conditions that are prone to hydrogen-induced cracking (HIC). This represents a huge risk when underwater wet welding is used for repair [5]. 

In Europe, the measurement of diffusible hydrogen in arc-welded metal is regulated by the ISO standard 3690 [6]. This standard is similar to the American AWS A4.3-93 [7] and the Japanese JIS Z 3113 [8] standards. There are differences in detail; however, with respect to the described methods, the standards are equivalent for the most part. For the present study, DIN EN ISO 3690:2018-12 [9] is used as the reference standard.

Within the last 35 years, different research groups all over the world measured diffusible hydrogen in wet weldments [1,10,11,12,13,14,15,16,17,18,19,20,21]. Most of these groups based their methods on one of the above-mentioned standards. However, many aspects like the sample geometry, analyzing time, analyzing method, and analyzing temperature that were used differed in many of the studies or were not stated at all. Thus, the main objective that led to the present study was to determine the influence that these differences might have on the hydrogen concentration and the comparability of values published in articles since 1983.

In 1998, some of the regulations of ISO 3690 [6] were evaluated in a European study on dry welding [22]. It was revealed that the sample preparation (namely, belt grinding and sanding), greasy surfaces, and the time span between the extinction of the arc and cooling in water (before storage in liquid nitrogen, to slow down hydrogen diffusion inside the samples) have an influence on the diffusible hydrogen content. No influence could be found regarding the temperature of the cooling water (between 5 °C and 60 °C, no difference was seen in the data), the base materials (S355 and S235 were compared), and the sample dimensions. The mean hydrogen contents determined for different sample dimensions were similar, while only the variance within the results was different. This could be due to lower and, thus, harder-to-detect hydrogen amounts in smaller samples [22]. 

DIN EN ISO 3690:2018 describes four methods to measure the diffusible hydrogen in welded samples. Three of these methods are recommended [9]:

- Mercury method (HG): The hydrogen is collected in a Y-tube over a liquid in which it will not dissolve, usually mercury, at 25 °C ± 5 °C.
- Measurement with a thermal conductivity detector (TCD):
  ⚬ Carrier gas hot extraction (CGHE) method: Hydrogen is extracted within a short period of time at a (suggested) temperature of 400 °C. The hydrogen is continuously collected and measured until all the diffusible hydrogen is quantified.
  ⚬ Long-term desorption (LTD) method: The sample is placed in a suitable container, which is purged with inert gas and sealed against the atmosphere. The collection of hydrogen is done at lower temperatures (usually 45 to 100 °C) for longer time spans (as compared to CGHE).
- Glycerin method: This method works like the mercury method, but uses glycerin, which is not toxic. However, the method is not recommended to be used, since glycerin can solve hydrogen.

Regarding these methods, studies showed that the TCD method used at lower temperatures in closed vessels for long-term desorption of hydrogen yields similar results to the mercury method (HG) [22,23,24,25,26,27]. The hydrogen concentrations determined by the LTD method and the HG method were found to be higher than those obtained with the glycerin method. However, this difference is less, if the hydrogen concentration is higher [24,27]. This can be explained by the solubility of hydrogen in glycerin. It was also implied that the LTD method might not require the use of liquid nitrogen for sample preparation, and that it shows less variance in the results than the mercury method, while taking less measurement time due to the higher analysis temperature [24]. Regarding the measurement time, it was stated that the minimum gas collection time at a temperature of 45 °C should be 48 h. At a temperature of 60 °C, a period of 24 h was regarded as appropriate for the collection of diffusible hydrogen [24]. These periods are shorter than those stated in DIN EN ISO 3690:2018 (72 h at 45 °C and more than 36 h at 60 °C) [9].

All these studies only focused on rather small hydrogen contents of samples welded in dry atmosphere. The largest diffusible hydrogen content measured in the European study [22] was 12 mL/100 g weld metal. For underwater wet-shielded metal arc welding (SMAW), much higher values 7 are expected. 

Thus, the present study examined the below specifications made in DIN EN ISO 3690 [9].

### 1.1. Normalization of Diffusible Hydrogen Content

**Figure 1:**
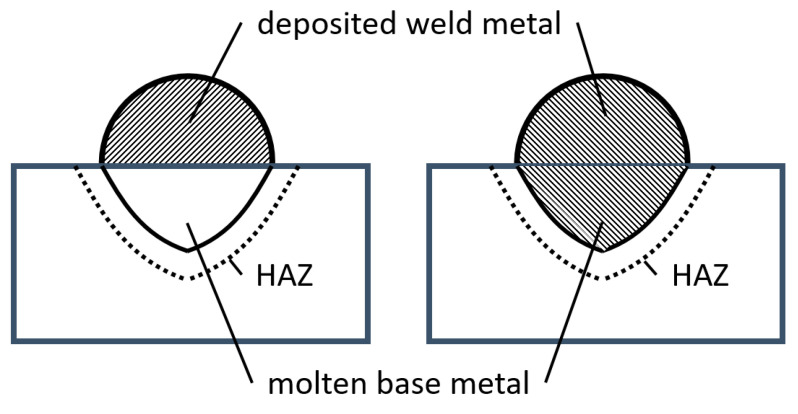
Schematic view of a weld bead micro section: the hatched areas represent the volume of molten material used for normalizing the effused hydrogen; left: deposited weld metal only (*H*_D_), right: deposited weld metal and molten base metal (*H*_F_).

Since hydrogen effuses in gaseous form, the equivalent to the intensity of the signal of the thermal conductivity detector is [volume] = mL. Of course, larger samples will store more diffusible hydrogen than small ones. Thus, a comparison is only meaningful if the results are normalized. DIN EN ISO 3690:2018 [9] proposes two ways of normalization for diffusible hydrogen contents:

1. The diffusible hydrogen content *H*_D_ represents the effused volume of hydrogen normalized by the weight difference of the specimen before and after welding. This means that the referenced mass is the deposited weld metal (Figure 1) and, thus, the unit of *H*_D_ is mL/100 g of weld metal.
2. The diffusible hydrogen content *H*_F_ represents the effused volume of hydrogen normalized by the total mass of the molten material (Figure 1). This includes both the molten base metal and the deposited weld metal. To use *H*_F_, the penetration depth of the weldment must be known. This means that etched micrographs of every sample need to be evaluated (ideally from both sides, to see changes in the penetration profile). The proposed unit of *H*_F_ is ppm (with respect to the mass of the molten material).

Typically, the conversion between ppm and mL/100 g is done using a simple factor (ppm = 0.9 × mL/100 g) [9], and DIN EN ISO 3690:2018 states an expected difference of 50% between *H*_D_ and *H*_F_. This is valid if the mass of deposited weld material is roughly the same as that of the molten volume of the base plate. Clearly, the difference between *H*_D_ and *H*_F_ depends on the ratio of deposited material to molten base material. At the same time, there are differences in penetration condition in underwater welding as compared to dry welding; thus, a difference between samples welded dry and wet has to be expected [20,28]. 

While *H*_F_ is clearly more accurate with respect to assessing the risk of HIC, the expenditure for its determination is also much higher. The differences in the normalization and their possible influences are, thus, important factors that were analyzed in the present study.

### 1.2. Number of Samples and Sample Dimensions

Three different sets of sample geometries are specified in the standard DIN EN ISO 3690:2018 [9]. Since both *H*_D_ and *H*_F_ are normalized to a reference mass, the size of the samples should not have any influence on the hydrogen content determined. This was already shown for small hydrogen contents [22]. However, for the amounts of diffusible hydrogen expected in wet welding, a verification was needed. 

Regarding the number of samples generated from one weld seam, DIN EN ISO 3690:2018 [9] does not recommend the usage of more than one sample at a time. However, the previous standard DIN 8572 [25] from 1981, allowed the use of up to four samples simultaneously. It had to be clarified which approach is suited for underwater wet welding. 

### 1.3. Time Periods Permitted for the Welding and Cleaning/Breaking Process

The time span between the extinguishing of the arc and the cooling in ice water for a valid experiment is specified as 4 s ± 1 s in the standard DIN EN ISO 3690:2018 [9]. Thier et al. identified this time span as a key variable, while the temperature of the water was not a critical factor [22]. In wet welding, this time period does not apply, since the sample is immersed in the water all the time. The influence of the time between the actual welding and the subsequent transfer into liquid nitrogen was not previously investigated, and it was, in addition to the cleaning time, part of this study. Since diffusion of hydrogen is rapid in ferritic steel at room temperature, an effect of this time span was expected.

### 1.4. Time, Temperature, and Method Defined for the Analysis

Salmi et al. [29] did research on hydrogen contents in 22MnB5 steel. They raised the temperature by 0.25 K/s from room temperature (RT) to 900 °C and found peaks in hydrogen content at 142, 341, and 513 °C. This and other studies intended to separate the diffusible and residual hydrogen contents. The term “residual hydrogen” refers to hydrogen that is trapped within the iron lattice at defects, phase boundaries etc., and, thus, is not able to diffuse at room temperature. After a critical activation energy is supplied, hydrogen can escape from traps and diffuse to other critical sites. Salmi et al. concluded that only the third peak (513 °C) represents residually stored hydrogen; thus, 400 °C (as stated in DIN EN ISO 3690:2018 [9]) is suited to measure only the diffusible hydrogen. Verification of that hypothesis for underwater wet weldments was another objective of the present study.

In addition to the temperature, the analysis time can be varied in analyses using the carrier gas hot extraction method with a thermal conductivity detector. The method measures the thermal conductivity of a carrier gas (nitrogen) flowing around the heated sample, which is affected by the hydrogen that leaves the sample. This thermal conductivity is compared to the thermal conductivity of the hydrogen-free carrier gas. The CGHE method usually operates at a temperature of 400 °C, and the measurement should—according to DIN EN ISO 3690:2018—span a period of 1200 s [9]. 

In addition, two of the TCD measurement methods described in DIN EN ISO 3690:2018 [9] were compared: CGHE and long-term desorption of hydrogen in closed vessels at lower temperatures using an external degassing unit. A comparison to the mercury method was not done since the comparability was already investigated [22,24,25,26,27], and, despite the fact that the mercury method is still mentioned in the standard, the method should no longer be used given the toxicity of mercury.

## 2. Materials and Methods 

### 2.1. Wet Welding Process

All samples were beaded on plate weldments, performed at a water depth of 0.5 m, using an arc voltage-controlled (AVC [30,31]) three-axis automated welding system, which allows welding samples in accordance with all the regulations given in DIN EN ISO 3690:2018 [9], including the transfer of the samples into liquid nitrogen within 20 s of the arc extinguishing.

The welding parameters were as follows:

Welding power source: AMT 400 E-UW (AMT GmbH, Aachen, Germany);
Electrode: Aquaweld (DIN 2302-E 38 0 Z RB 2 UW 20 fr [32]) (Kjellberg Finsterwalde Elektroden und Zusatzwerkstoffe GmbH, Finsterwalde, Germany);
Target arc voltage: 30 V;
Welding current: 160 A;
Welding speed: 0.20 m/min;
Water depth: 0.5 m;
Polarity: direct current (DC) minus;
Base metal: S235JR (polished).

### 2.2. Normalization of the Measured Hydrogen Concentration

Macro cross-sections of welding samples were etched with 2% nital (in accordance with DIN EN ISO 17639:2013 [33]) and evaluated using a stereomicroscope (MZ 8 by Leica Microsystems GmbH). The areas of dilution were determined to find the ratio of deposited weld metal to molten base metal. 

**Figure 2:**
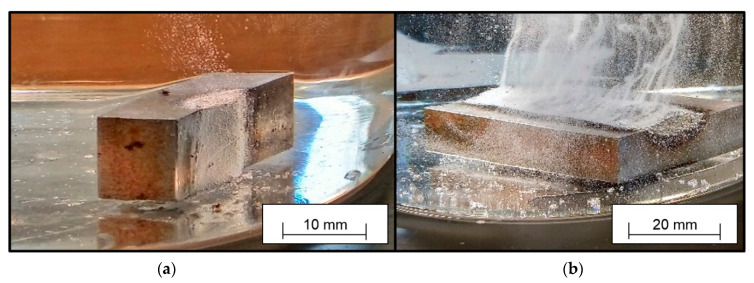
Samples outgassing in paraffin. Sample geometry (**a**) S1 at the beginning of the outgassing, and (**b**) S3 after 1200 s of outgassing.

In order to provide material where hydrogen is stored (and released during the analyses), samples were welded and cooled in accordance with the specifications of DIN EN ISO 3690:2018 [9]. Next, they were inserted into a heated glass bowl filled with paraffin oil at *T* = 100 °C. The elevated temperature accelerates the diffusion processes within the sample. Once the hydrogen leaves the sample, the high viscosity of the paraffin oil slows down the hydrogen transport, and the hydrogen is visible in form of small ascending bubbles (Figure 2).

### 2.3. Welding of the Samples

**Figure 3:**
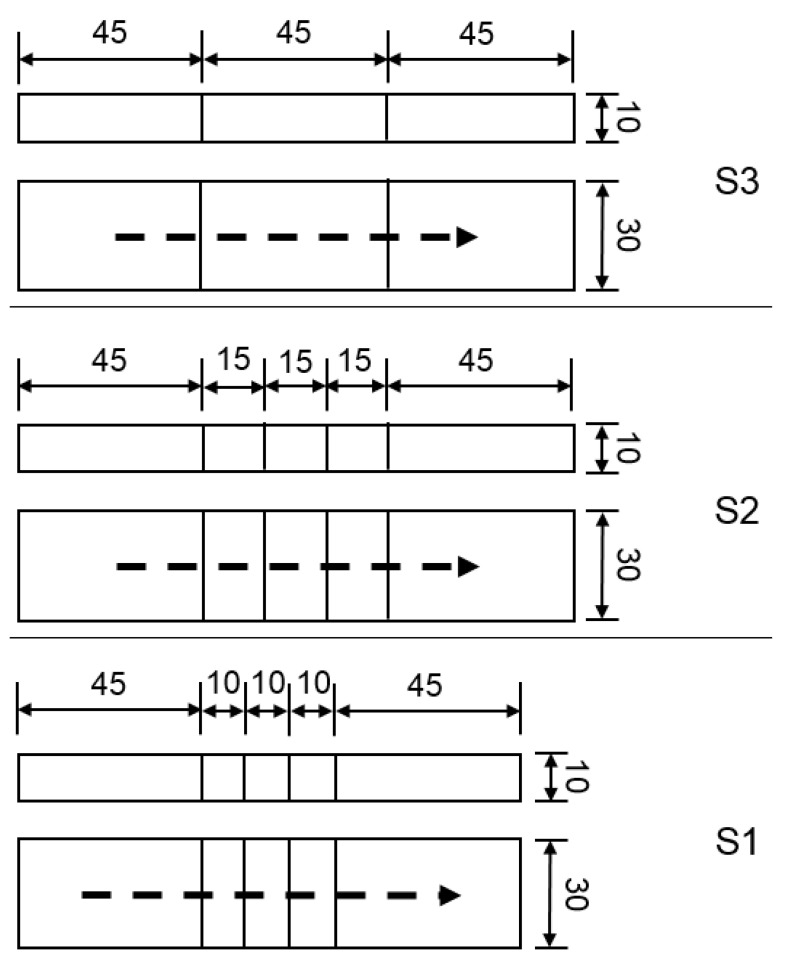
Schematic overview of the sample geometries used (each side view is shown above the respective top view); top to bottom: S3, S2, S1 (all lengths in mm).

Three different sample dimensions were welded to determine if the sample geometry has any influence on the diffusible hydrogen content *H*_D_. The sample dimensions used were based on DIN EN ISO 3690:2018 [9] with a sample size C of 15 mm × 10 mm × 30 mm (hereinafter referred to as S2). The length of the samples was varied from 10 mm (S1) to 45 mm (S3) (Figure 3). Thirty samples of each size were analyzed. The other geometries provided in DIN EN ISO 3690:2018 [9] were not considered. The sample geometries used in the present study offer the possibility to easily recognize effects depending on the proportion of sample surface to sample mass. 

To evaluate the influence of the number of samples welded simultaneously, three samples of the type S1 and S2 were welded in one welding step. All samples were marked individually and tested for differences in the diffusible hydrogen content correlating to their individual position in the weld seam (first, second, and last to be welded).

### 2.4. Time Limitations Defined Regarding the Welding and Cleaning Process

The standard DIN EN ISO 3690:2018 [9] allows 4 s ± 1 s after the arc is extinguished to place the welded sample into ice water. After another 20 s ± 2 s, the sample needs to be deposited into a freezing bath containing methanol, dry ice (solid CO_2_), or liquid nitrogen, to curtail diffusion processes. The temperature should, thus, be at least below −78 °C.

For wet welding processes, since the samples are already welded underwater, and the temperature of the water was found to not influence the results [22], the time span before deep freezing could be combined, and a minimum time span of 20 s from the extinction of the arc to the deposition of the specimen into the liquid nitrogen was used in the present study. In addition, this time span was varied in three steps: 20 s (i.e., based on DIN EN ISO 3690:2018 [9]), 120 s, and 300 s. For each time span, nine samples were analyzed. 

Following the DIN EN ISO standard 3690:2018 [9], further treatment of the samples was carried out. The maximum time span for the preparation of the samples for hydrogen measurement (sample separation and cleaning) was 60 s. Afterward, the samples were cooled for another 120 s before finishing the work. According to DIN EN ISO 3690:2018 [9], this is valid for samples that are cooled to −78 °C (dry ice temperature). Thus, the time needed to raise the sample temperature to −78 °C, after cooling in liquid nitrogen (−196 °C), was determined. For this purpose, the samples were taken from the liquid nitrogen after 120 s of cooling. Thereafter, they were left in a dry air atmosphere. The sample temperature was measured on the specimen surface with type K thermocouples, and the ambient temperature was held constant at 22 °C. The time was recorded and stopped when the sample’s surface reached −78 °C.

### 2.5. Time, Temperature, and Method Defined for the Analysis

The standard DIN EN ISO 3690:2018 [9] gives a table of temperature/time combinations suitable for the analysis of diffusible hydrogen. These temperature/time combinations are intended to ensure that the entire amount of diffusible hydrogen diffuses out of the sample. In the present study, the analyses were carried out using a hydrogen analyzer (G4-Phoenix by Bruker AXS GmbH, Karlsruhe, Germany) using the CGHE/TCD method. The temperature was firstly programmed as a ramp function rising from 50 to 900 °C (0.25 K/s, based on the methods described by Salmi et al. [29]), in order to differentiate between diffusible and residual hydrogen and to determine the critical temperature for the release of the residual hydrogen in order for the later experiments to record only the diffusible fraction as the measured quantity.

Afterward, isothermal analyzes were carried out within 1200 s at a temperature of 400 °C as described in DIN EN ISO 3690:2018 [9]. These results were compared to analysis with durations of 1800 s (for sample size S1), 2700 s (S2), and 3600 s (S3) at 400 °C. 

Additional samples were analyzed using the LTD method. A difference was expected, but this difference was expected to be small. Thus, it was decided to use a variety of different stick electrodes, providing a larger range of hydrogen concentrations. This was meant to improve the detection of differences within the expected variances. To compare the measuring methods, the weld metal of seven commercially available stick electrodes was analyzed using both the CGHE and the LTD methods. For both methods, the same kinds of welding samples (S2) were used. The measurements were performed using a thermal conductivity detector to measure hydrogen content in a carrier gas. In contrast to CGHE, the LTD samples were stored in collecting vessels at moderate temperatures below 100 °C for longer time spans. For the present study, 45 °C and 72 h were chosen. The welding speed and current were held constant at 0.2 m/min and 160 A, respectively. The target voltage was adjusted in order to obtain the best welding results for each stick electrode. For each electrode, nine samples were analyzed with each method. The electrodes used are listed in Table 1. 

## 3. Results

### 3.1. Normalization of the Hydrogen Concentration

The measurements of the weld penetration depth of five etched samples showed a mean ratio of molten base material to deposited weld material of 1 to 0.59 (over 1.7 times more molten base metal than deposited weld metal). 

The hydrogen measurement of 26 samples (S2) resulted in mean values of *H*_D_ = 65.98 mL/100 g and *H*_F_ = 24.21 mL/100 g. The difference was 63%. 

**Figure 4:**
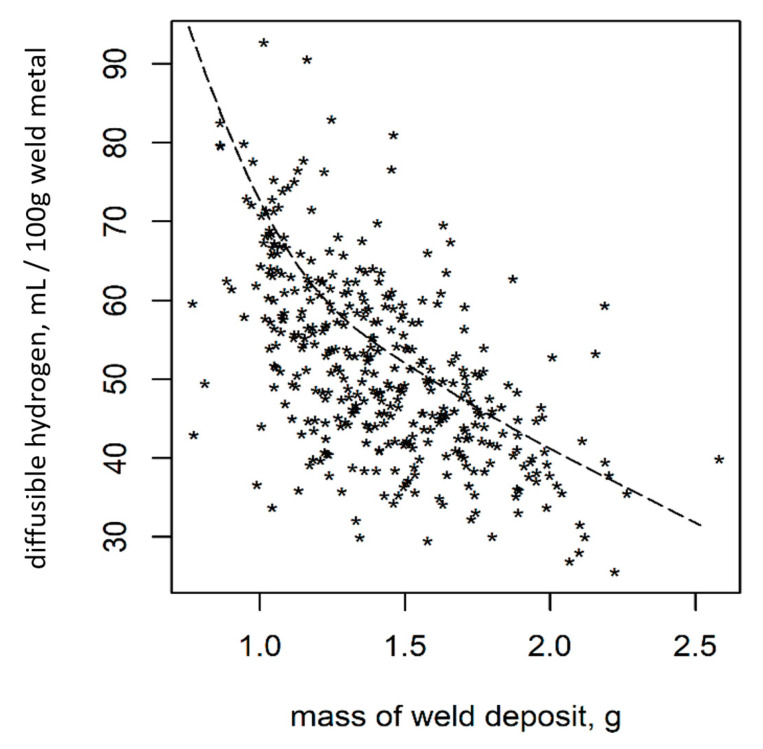
Correlation of *H*_D_ and the mass of deposited weld metal [31].

As shown previously [31], the hydrogen analysis shows a significant correlation of *H*_D_ to the mass of the deposited weld material (Figure 4). 

The visual observation of welded samples outgassing in highly viscous paraffin showed that the hydrogen not only diffused from the surface of the deposited weld material but also from the workpiece surface that was in a molten state during weldment (Figure 2). Gas bubbles emerged from the complete weld metal surface (deposited and molten base metal) and from the HAZ. The molten base metal was only considered for the standardization of *H*_F_, not for *H*_D_.

### 3.2. Time Limitations Defined Regarding the Welding and Cleaning/Breaking Process

DIN EN ISO 3690:2018 [9] limits the time to separate and clean the samples to 60 s. Thereafter, 120 s of freezing is stated to be necessary in order not to bias the results. Since the standard allows the usage of dry ice for freezing, the time it takes for a sample coming from −196 °C to reach −78 °C, while being exposed to room temperature (22 °C), was measured in order to define time limits for the storage in liquid nitrogen. The experiment demonstrated that 440 s were needed until the sample’s temperature increased to −78 °C. 

**Figure 5:**
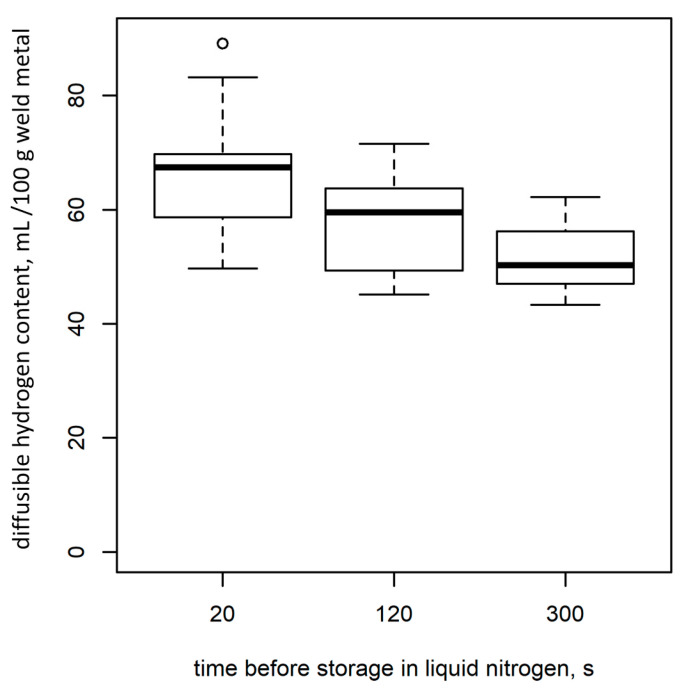
Influence of the time span between welding and storage in liquid nitrogen on the diffusible hydrogen content *H*_D_. Displayed as a Boxplot: the horizontal line within the boxes represents the median, the boxes represent the interquartile range. The whiskers show the maximum or minimum of each distribution; outliners are marked with an “o”.

Figure 5 shows the effect of the time span between the extinguishing of the arc and the deposition in liquid nitrogen for the three periods of 20 s, 120 s, and 300 s. The differences in mean values of *H*_D_ were 7.7 mL/100 g (11.7%) from 20 s to 120 s and 14.3 mL/100 g (21.7%) from 20 s to 300 s. All results were tested for equality and determined as significantly different (test: Analysis of variance (ANOVA), *p* < 0.05).

### 3.3. Analyses Regarding the Sample’s Dimensions and the Number of Samples Welded Simultaneously

**Figure 6:**
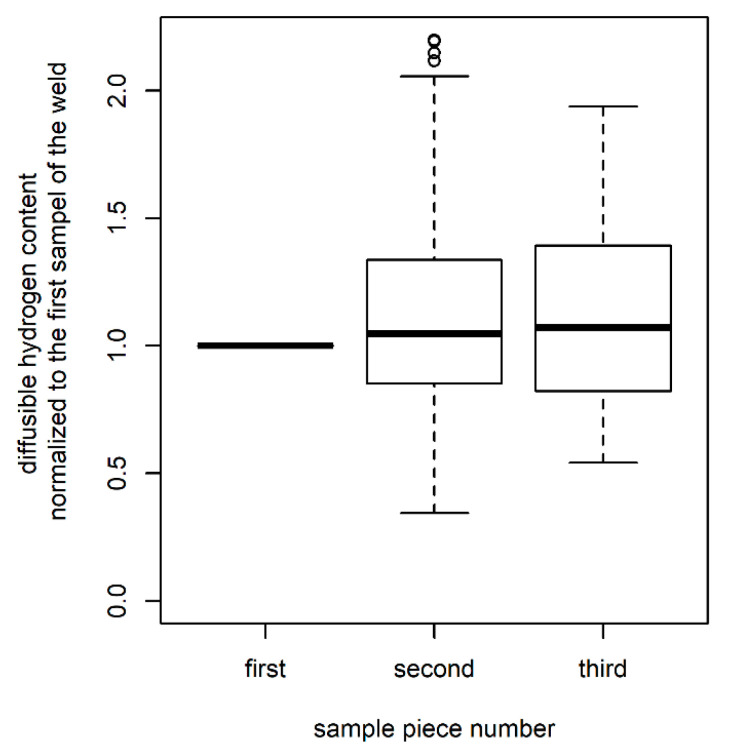
Boxplot showing the diffusible hydrogen concentration normalized with respect to the first welded sample; data are for all samples with the dimensions S1 and S2.

To evaluate if the simultaneous weldment of three samples corrupts the results, all samples of S1 and S2 were characterized. Since the samples were marked regarding their position in the weld seam (first, second, and third to be welded over), normalization was the first step. Therefore, the first sample of each weld seam was taken as the reference. The hydrogen contents of the second and third samples were then referenced to this value. This led to values bigger than 1 if the sample contained more diffusible hydrogen than the respective first welded sample and to values lower than 1 if the diffusible hydrogen content of the sample was lower than the hydrogen content in the first sample of the weld seam. The results are shown in Figure 6. While some samples showed rising *H*_D_ values from the first to the last sample, others showed the opposite trend. Hence, the results of all samples were tested for differences (ANOVA). Due to the large variance, no significant trend could be identified. It was, therefore, concluded that (at least for these data) the evaluation of three samples welded at once is valid. 

**Figure 7:**
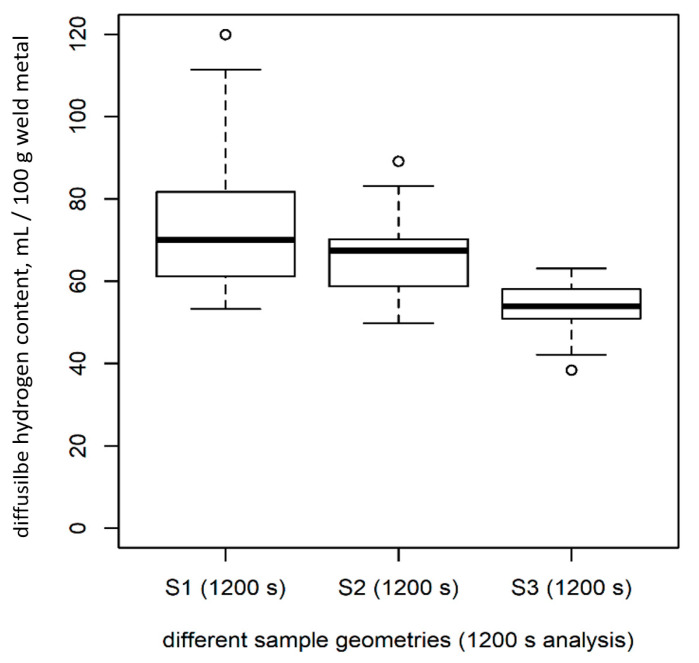
Correlation of sample geometries and *H_D_* for an analysis time of 20 min at 400 °C.

For each sample length (S1, S2, and S3), *n* = 30 samples were analyzed for 1200 s at 400 °C using the CGHE/TCD method. There were significant differences in the amount of diffusible hydrogen measured in samples of different lengths (Table 2, Figure 7; test: ANOVA, *p* < 0.05), although the samples were standardized regarding the weight of the deposited weld material (*H*_D_) and, thus, should have been similar. 

**Figure 8:**
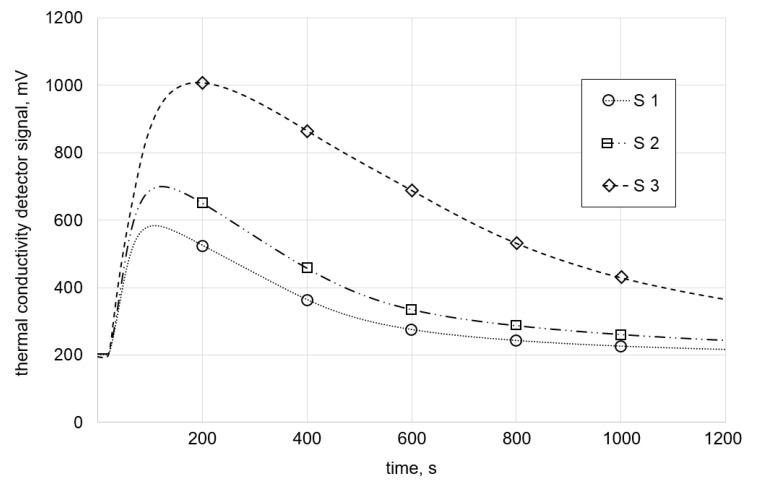
Measured hydrogen content within 1200 s (20 min). None of the graphs perfectly meet the base line after this time span.

A closer look at the graphs of the measured hydrogen signal over time (Figure 8) reveals that the 1200-s period was not enough time to degas all stored diffusible hydrogen in large samples. 

This directly led to the next point of interest, i.e., the time used for the analysis. To determine suitable analysis periods, the curves of the signals in Figure 8 were extended asymptotically. Next, the intersection with the baseline was determined, and the graphically determined periods were then employed in actual measurements. The results are shown in the next section. 

### 3.4. Time, Temperature, and Methods Defined for the Analysis

**Figure 9:**
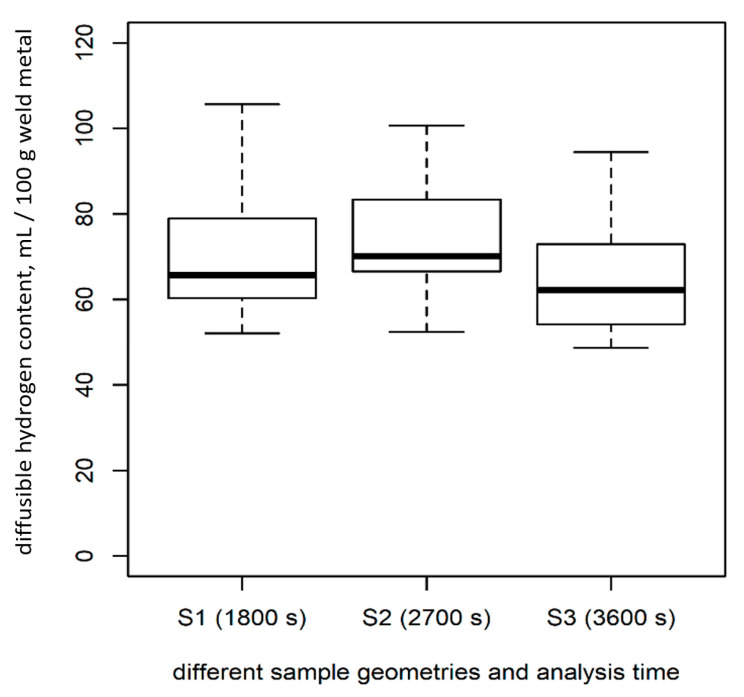
Effect of variation of the analysis time for the individual sample geometries on the diffusible hydrogen content *H*_D_.

For the CGHE method, when the analysis time was extended to 1800 s (S1), 2700 s (S2), and 3600 s (S3) (each *n* = 9), instead of 1200 s, all signals reached their individual starting point (base line). If the time was chosen accordingly, no significant differences in *H*_D_ could be found between the samples of varying geometry (Figure 9 compared to Figure 7; test: ANOVA, *p* > 0.1).

For sample geometry S1, there was no difference in the mean values of *H*_D_ measured for 1200 s and 1800 s of analyzing time (Figure 7 and Figure 9). The difference in the mean value of *H*_D_ regarding the short (1200 s) and the long analysis time (2700 s and 3600 s) for the larger samples was significant. For the medium-sized samples (S2), the difference was 14.9%, and, for the long samples (S3), the difference was 21.1%, as compared to the short measuring time (Figure 7 compared with Figure 9). 

**Figure 10:**
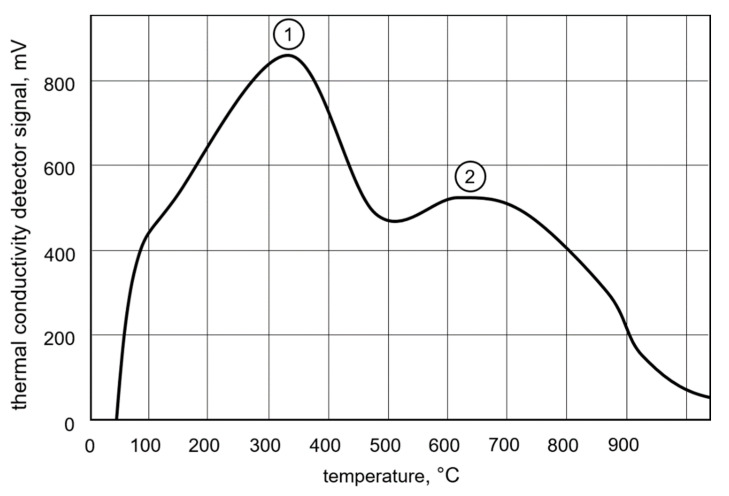
Signal of the thermal conductivity sensor while raising the temperature from 50 to 900 °C; point 1 marks the peak for the diffusible hydrogen, while point 2 marks the peak for the residual hydrogen.

The result of the investigation regarding the analysis temperature is shown in Figure 10. The graph shows the signal of the thermal conductivity sensor, recorded within one hour of heating while raising the temperature from 50 to 900 °C. There were two peaks visible. The first occurred at 330 °C (Figure 10, Point 1) and a smaller one occurred at 620 °C (Figure 10, Point 2). 

**Figure 11:**
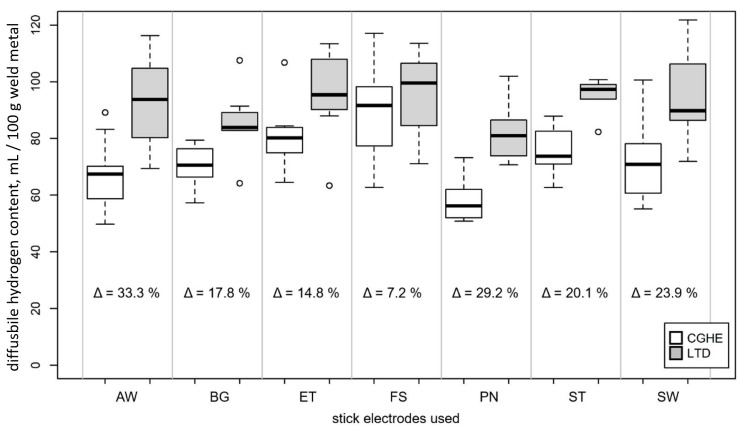
Comparison of the two different analyzing methods for different stick electrodes, using sample geometry S2 (electrode designations and parameters are shown in Table 1).

The results of the comparison between the two measurement methods of LTD and CGHE (both measured using the same TCD) are shown in Figure 11. The average difference between the mean *H*_D_ values of the two methods was 19.3 mL/100 g weld metal. For all electrodes investigated, the mean value of the diffusible hydrogen content measured with the LTD method was higher than the mean value of measurements with the CGHE method. The mean difference between the LTD and the CGHE method was 20.9%. 

## 4. Discussion

### 4.1. Normalization of the Measurement Hydrogen Concentration

Measurements of the penetration depth for wet welded samples showed that the ratio of molten base material to deposited weld material is not one to one (1:1) as supposed in the standard DIN EN ISO 3690:2018 [9]. Thus, *H*_F_ is not 50% of the value of *H*_D_. This is in accordance with the results of Zhang et al., who investigated weld bead geometries in wet welding [28]. There was a correlation between the mass of deposited weld material and the values of *H*_D_ (Figure 4 [31]). This can be explained by looking at the follow-up experiment, where the diffusion out of the welded samples was observed (Figure 2). It can be seen that hydrogen bubbles were formed at the primarily molten base material, as well as at the deposited weld material. By only including the deposited weld material in the normalization (as done for *H*_D_), irregularities in the weldments had a huge influence. If the volume of molten base material stays the same, but the volume of deposited weld material varies, the considered mass for *H*_D_ varies much more than the actual metal volume, which stores and later, during analysis, releases the hydrogen. The higher variance of irregularities and unsteadiness of weld bead geometries in wet welding compared to dry welding was also reported in earlier research [28,34]. This explains the significant correlation between the mass of deposited weld metal and *H*_D_. Including the mass in multivariate correlation models is a valid method to account for this influence [31]. 

### 4.2. Time Limitations Defined Regarding the Welding and Cleaning/Breaking Process

The time span between the extinguishing of the arc and the deposition in liquid nitrogen is critical for any analysis of diffusible hydrogen content and, thus, should be held constant. A difference in this time span from 20 to 120 s can reduce the mean measured diffusible hydrogen content *H*_D_ by over 11%. Expanding the duration to 300 s resulted in an almost 22% reduced mean *H*_D_ value (Figure 5). Unless special measures are taken, this means that samples welded manually in larger water depths will inescapably lead to lower measured hydrogen values due to the time needed for the sample to reach the water’s surface and the liquid nitrogen. Clearly, for meaningful comparison of data, it is essential to specify the time which elapses from the extinction of the arc to the sample’s insertion into the cooling medium. 

The cleaning time on the other hand should have little influence on the results if liquid nitrogen is used, and if the time does not exceed 420 s. However, it was not investigated whether the diffusion of hydrogen out of the sample is reduced upon using a lower temperature of liquid nitrogen instead of dry ice.

### 4.3. Sample Dimensions and Time for Analysis

The results regarding the sample dimensions show that there was less variance in *H*_D_ when analyzing larger samples (Table 2). This is in accordance with the results reported in Reference [19]. This can be attributed to the fact that larger samples portray a larger variety of welding inhomogeneity in one sample. Thus, inhomogeneity has less influence on the measured *H*_D_ value. The variance in the mass of deposited weld metal is also consequently less. To obtain reasonable results when using the CGHE method, the heating time has to be longer for larger samples (Figure 7 and Figure 9). For large samples (S3), at least 3600 s of analyzing should be used. For samples S2 (DIN EN ISO 3690:2018 [9] sample size C), at least 30 min instead of 20 min is preferable in order to measure the whole amount of diffusible hydrogen produced by the wet welding process. 

### 4.4. Analysis Temperature for CGHE

The temperature of 400 °C (as stated in DIN EN ISO 3690:2018 [9]) turned out to be suitable for the materials and processes used in the present study. The first peak recorded when measuring with rising temperature could be associated with the diffusible hydrogen, while the residually stored hydrogen left the material at around 600 °C (Figure 10). These results are similar to those obtained in earlier studies [29,35]. The slight difference in peak temperatures can be attributed to the difference in the materials welded, which in turn affect the type and distribution of the hydrogen traps present.

### 4.5. Analysis Method

The analysis method used can influence the measured hydrogen content and, thus, should always be indicated. The two methods investigated in the present study (CGHE and LTD) showed a mean difference of 20.6% in measured *H*_D_ values. The fact that the contents measured at 45 °C (LTD) were higher than the values measured at 400 °C (CGHE) cannot be connected to residual hydrogen. In this case, the results of the CGHE method would be higher than those of the LTD method. Figure 10 also illustrates that no residual hydrogen was measured at 400 °C.

**Figure 12:**
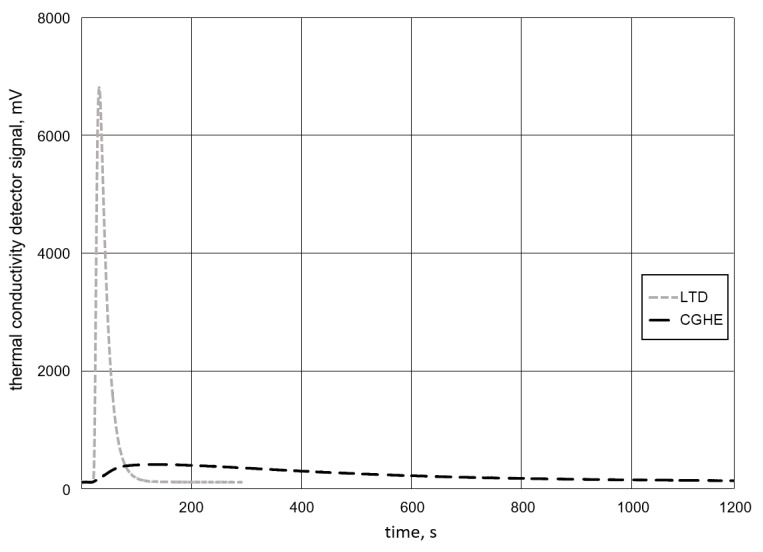
Comparison of the signals of the thermal conductivity detector for the methods of long-term desorption (LTD) and carrier gas hot extraction (CGHE).

The difference could be due to the fact that the LTD method stores all the hydrogen diffusing out of the sample in a vessel. The whole amount is then measured at once. In contrast, the CGHE method measures smaller amounts of gas continuously over the analyzing time (Figure 12). Since there is a minimum detection limit, some amount of the analyzed gas is more likely to be below this limit and get lost in the process. This source of error is clearly smaller for the LTD measurement. 

For comparative studies, the method used has no influence. Considering the mean variation of the hydrogen content, the qualitative trends of the mean values remain the same for both methods.

### 4.6. Recommendations

For future studies, the use of samples with the dimensions of S2 (ISO 3690:2018 sample C) and 1800 s of analysis time at 400 °C using CGHE/TCD is recommended. The other sample dimensions showed a higher mean variation (see sample S1) or led to an extended analyzing time (see sample S3). Three samples can be welded at once, but every sample then needs be marked to reveal effects of the individual position of each sample. 

The samples should be stored in liquid nitrogen after 20 s, or the time span should be stated if it exceeds the regulations of DIN EN ISO 3690:2018 [9]. The time used to clean and separate the samples should not exceed 420 s (7 min) before further cooling. For normalization, all areas that were molten during the welding process should be considered. The use of *H*_F_ is, thus, preferred, although *H*_F_ requires many measurements. If *H*_D_ is to be used, a confidence interval regarding the sample’s mass should be calculated, and samples with too little or too much mass should be excluded. Furthermore, for comparative studies, the inclusion of the mass as a variable in a multivariate fitting model should be considered in order to not confuse the influence of the mass with other influences. This is especially important when parameters like arc voltage, welding current, and welding polarity are compared, since these parameters can change the weld bead geometry, as well as the ratio of molten base material and deposited weld material [34,36]. 

There are many factors influencing the diffusible hydrogen content in underwater wet welding. The variance in the measured values is high compared to dry welding. To obtain meaningful results despite these difficulties, the standards for the measurement of diffusible hydrogen should be high and consistent. Only strict compliance to the times, temperatures, methods, and sample preparations can provide the data needed to assess the HIC risk in wet welding. 

## 5. Conclusions

In the present study, the hydrogen content analysis procedure specified in the standard DIN EN ISO 3690:2018 [9] was investigated regarding the applicability to underwater wet welding.

The main results can be summarized as follows:

1. The time between the extinguishing of the arc and cooling in liquid nitrogen significantly influences the measured diffusible hydrogen content in welded samples.
2. For DIN EN ISO 3690:2018 [9] sample C, up to three samples can be taken simultaneously in one welding step without significantly affecting the results.
3. Different sample geometries need different timespans to release all the diffusible hydrogen during measurement. For DIN EN ISO 3690:2018 [9] sample C, 30 min at 400 °C is suitable.
4. The hydrogen analysis methods using thermal conductivity detectors (long-term desorption and carrier gas hot extraction) differ in results. Long-term desorption leads to lower mean values.

## Figures and Tables

**Table 1:** Electrodes used for the comparison of the analyzing methods.

Manufacturer	Designation of Electrode	Target Voltage	Abbreviation
Hydroweld Ltd. (Sutton Coldfield, UK)	Hydroweld FS	28 V	FS
Broco Inc. (Ontario, CA, USA)	Broco EasyTouch	28 V	ET
Broco Inc. (Ontario, CA, USA)	Broco SoftTouch	28 V	ST
ESAB AB (Göteborg, Sweden)	Arcair Sea-Weld	33 V	SW
Speciality Welds Ltd. (Rawfolds, UK)	Barracuda Gold	31 V	BG
Voestalpine Böhler Welding GmbH (Düsseldorf, Germany)	Phoenix Nautica 20	30 V	PN
Kjellberg Finsterwalde Elektroden und Zusatzwerkstoffe GmbH (Finsterwalde, Germany)	Aquaweld	30 V	AW

**Table 2:** Diffusible hydrogen contents in mL/100 g weld metal of samples of three different dimensions (analyzed for 1200 s at 400 °C).

	Mean Value	Variance
S1	71.75	16.65
S2	66.21	9.65
S3	54.13	5.93

## References

[1] Hassel T. (2013). Systematische Untersuchung von Lichtbogenprozessen für das Nasse Elektrodenschweißen unter Wasser in Tiefen Größer 20 Meter: Abschlussbericht zu IGF-Vorhaben 16777 N. Forschungsvereinigung Schweißen und verwandte Verfahren e.V. des DVS.

[2] Lippold J.C. (2014). Welding Metallurgy and Weldability.

[3] Deutscher Verband für Schweisstechnik e.V (2017). Merkblatt DVS 1818—Ausführung von Lichtbogenschweißarbeiten in Nasser Umgebung.

[4] Yushchenko K.A., Gretskii Y.Y., Maksimov S.Y. (1997). Study of physicometallurgical peculiarities of wet arc welding of structural steels. Proceedings of the Underwater Wet Welding and Cutting, International Seminar and Workshop.

[5] Fydrych D., Łabanowski J., Rogalski G. (2013). Weldability of high strength steels in wet welding conditions. Pol. Marit. Res..

[6] ISO–International Organization for Standardization (2018). ISO 3690:2018-07: Welding and Allied Processes – Determination of Hydrogen Content in Arc Weld Metal.

[7] AWS–American Welding Society (1993). ANSI/AWS A4.3-93:R2006:1993: Standard Methods for Determination of the Diffusible Hydrogen Content of Martensitic, Bainitic, and Ferritic Steel Weld Metal Produced by Arc Welding.

[8] JIS–Japanese Standard Association (1975). JIS Z 3113:1975: Method for Measurement of Hydrogen Evolved from Deposited Metal.

[9] DIN—Deutsches Institut für Normung e. V (2018). DIN EN ISO 3690:2018-12: Schweißen und Verwandte Prozesse—Bestimmung des Wasserstoffgehaltes im Lichtbogenschweißgut.

[10] Ando S., Asahina T., Ando S., Asahina T. (1983). A Study on the Metallurgical Properties of Steel Welds with Underwater Gravity Welding. Underwater Welding, Proceedings of the International Conference, Trondheim, Norway, 27–28 June 1983.

[11] Bartzsch J. (2002). Untersuchungen zu Metallurgischen und Physikalischen Vorgängen beim Schweissen unter Extremen Bedingungen.

[12] Pitrun M., Nolan D., Dunne D. (2004). Diffusible hydrogen content in rutile flux-cored arc welds as a function of the welding parameters. Weld. World.

[13] Da Silva W.C.D., Ribeiro L.F., Bracarense A.Q., Pessoa E.C.P. (2012). Effect of the Hydrostatic Pressure in the Diffusible Hydrogen at the Underwater Wet Welding. Proceedings of the ASME 31st International Conference on Ocean, Offshore and Arctic Engineering.

[14] Świerczyńska A., Fydrych D., Rogalski G. (2017). Diffusible hydrogen management in underwater wet self-shielded flux cored arc welding. Int. J. Hydrog. Energy.

[15] Fydrych D., Świerczyńska A., Rogalski G. (2015). Effect of Underwater Wet Welding Conditions on the Diffusible Hydrogen Content in Deposited Metal. Metall. Ital..

[16] Kong X., Li C., Zou Y., Zhang J., Hu Y., Wang J., Qaddoumi N., Koh S.-K., Devlin J. (2016). Measurement and Analysis of the Diffusible Hydrogen in Underwater Wet Welding Joint. MATEC Web Conf..

[17] Kussike S.M. (2015). Hydrophobierung von Stabelektroden für das “nasse” Lichtbogenhandschweißen unter Wasser. Ph.D. Thesis.

[18] Garašić I., Kralj S., Kožuh Z. (2009). Investigation into cold cracking in underwater wet welding of API 5L X70 steel. Trans. FAMENA.

[19] Gooch T.G. (1983). Properties of underwater welds. Part 1: Procedural Trials. Metal. Constr..

[20] Hamkens J.H. (1991). Unterwassernaßschweißen mit selbstschützenden Fülldrahtelektroden. Ph.D. Thesis.

[21] Fydrych D., Rogalski G. (2013). Effect of underwater local cavity welding method conditions on diffusible hydrogen content in deposited metal. Weld. Int..

[22] Thier H., Eisenbeis C. (1998). Schweißen des Stahls S 355 J 2 G 3(Fe E 355) ohne Vorwärmung—Ermittlung einer Meßmethode zur schnellen und zuverlässigen Bestimmung sehr kleiner Wasserstoffgehalte. Forschungsbericht der Europäischen Kommission.

[23] Sievers E.R., Müller L. (2007). Laserschweißen Innovativer, Hochfester Stähle unter dem Aspekt Wasserstoff und Rissanfälligkeit (LaHRissa).

[24] Abreu L.C., Modenesi P.J., Villani-Marques P. (1995). Comparative study of methods for determining the diffusible hydrogen content in welds. Weld. Int..

[25] DIN—Deutsches Institut für Normung e. V (1981). DIN 8572:1981-03: Bestimmung des diffusiblen Wasserstoffs im Schweissgut.

[26] Kannengiesser T., Tiersch N. (2010). Comparative Study between Hot Extraction Methods and Mercury Method—A National Round Robin Test. Weld. World.

[27] Fydrych D., Łabanowski J. (2012). Determining diffusible hydrogen amounts using the mercury method. Weld. Int..

[28] Zhang Y., Jia C., Zhao B., Hu J., Wu C. (2016). Heat input and metal transfer influences on the weld geometry and microstructure during underwater wet FCAW. J. Mater. Process. Technol..

[29] Salmi S., Rhode M., Jüttner S., Zinke M. (2015). Hydrogen determination in 22MnB5 steel grade by use of carrier gas hot extraction technique. Weld. World.

[30] Hecht-Linowitzki V., Klett J., Hassel T. Automated Underwater Arc Welding. Proceedings of the Symposium on Automated Systems and Technologies.

[31] Klett J., Hecht-Linowitzki V., Grünzel O., Schmidt E., Maier H.J., Hassel T. (2020). Effect of the water depth on the hydrogen content in SMAW wet welded joints. SN Appl. Sci..

[32] DIN–Deutsches Institut für Normung e. V (2018). DIN 2302:2018-03: Schweißzusätze -Umhüllte Stabelektroden zum Lichtbogenhandschweißen von unlegierten Stählen und Feinkornstählen in nasser Überdruckumgebung Einteilung.

[33] DIN—Deutsches Institut für Normung e. V (2013). DIN EN ISO 17639: Zerstörende Prüfung von Schweißverbindungen an Metallischen Werkstoffen—Makroskopische und Mikroskopische Untersuchungen von Schweißnähten.

[34] Hassel T., Hecht-Linowitzki V., Kussike S.M., Rehfeldt D., Bach F.-W. (2015). Systematic investigation into wet arc welding under water with covered stick electrodes. Weld. Cut..

[35] Escobar D.P., Depover T., Duprez L., Verbeken K., Verhaege M. (2012). Combined thermal desorption spectroscopy, differential scanning calorimetry, scanning electron microscopy and X-ray diffraction study of hydrogen trapping in cold deformed TRIP steel. Acta Mater..

[36] Gunarajan V., Murugan N. (2000). Prediction and optimization of weld bead volume for the submerged arc process Part 1. Weld. Res. Suppl..

